# The role of HLA-G in primary biliary cholangitis and response to therapy

**DOI:** 10.3389/fimmu.2025.1585535

**Published:** 2025-07-29

**Authors:** Michela Miglianti, Stefano Mocci, Roberto Littera, Giancarlo Serra, Cinzia Balestieri, Maria Conti, Francesco Pes, Silvia Deidda, Michela Lorrai, Caterina Mereu, Michela Murgia, Celeste Sanna, Alessia Mascia, Francesca Sedda, Irena Duś-Ilnicka, Selene Cipri, Mauro Giovanni Carta, Sara Lai, Erika Giuressi, Maurizio Melis, Teresa Zolfino, Sabrina Giglio, Andrea Perra, Luchino Chessa

**Affiliations:** ^1^ Department of Medical Sciences and Public Health, University of Cagliari, Cagliari, Italy; ^2^ Medical Genetics, Department of Medical Sciences and Public Health, University of Cagliari, Cagliari, Italy; ^3^ Centre for Research University Services (CeSAR, Centro Servizi di Ateneo per la Ricerca), University of Cagliari, Cagliari, Italy; ^4^ AART-ODV (Association for the Advancement of Research Transplantation), Cagliari, Italy; ^5^ Medical Genetics, R. Binaghi Hospital, ASL Cagliari, Cagliari, Italy; ^6^ Gastroenterology Unit, ARNAS Brotzu, Cagliari, Italy; ^7^ Pneumology Unit, R. Binaghi Hospital, ASSL Cagliari, Cagliari, Italy; ^8^ Oncology and Molecular Pathology Unit, Department of Biomedical Sciences, University of Cagliari, Cagliari, Italy; ^9^ Department of Oral Pathology, Wrocław Medical University, Wrocław, Poland; ^10^ Liver Unit, Department of Internal Medicine, University Hospital of Cagliari, Cagliari, Italy

**Keywords:** HLA-G, primary biliary cholangitis, ursodeoxicholic acid, Sardinia (Italy), autoimmune diseases

## Abstract

**Introduction:**

Primary biliary cholangitis (PBC) is a rare autoimmune liver disease involving bile duct damage and fibrosis. This study explores the role of HLA-G, an immunomodulatory molecule crucial for immune tolerance, in PBC pathogenesis and treatment.

**Methods:**

A cohort of 166 PBC patients from Sardinia was compared to 180 healthy controls and 205 autoimmune hepatitis type 1 (AIH-1) patients. Plasma soluble HLA-G (sHLA-G) levels, *HLA-G* alleles, and *3’UTR* haplotypes were analyzed alongside clinical data, including therapy response to ursodeoxycholic acid.

**Results:**

The UTR-1 haplotype was significantly more frequent in PBC patients than in controls (48.2% vs 34.3%, Pc= 0.0018). The extended haplotype HLA-G*01:01:01:08/UTR-1 was also strongly associated with PBC (23.2% vs 12.5% in controls, Pc = 0.008; 23.2% vs 6.6% in AIH-1, Pc= 2.6×10_-9_). PBC patients exhibited lower sHLA-G levels compared to controls and AIH-1 (9.1 U/mL vs 24.03 U/mL and 13.9 U/mL, respectively). Among *UTR-1* carriers, sHLA-G levels were particularly reduced in PBC patients. The HLA-G*01:01:01:08/UTR-1 haplotype correlated with the lowest sHLA-G levels and poorer therapy response (60% vs 24.1%, P = 0.0001).

**Discussion:**

These findings suggest HLA-G variants, especially HLA-G*01:01:01:08/UTR-1, as potential biomarkers for PBC prognosis and treatment outcomes.

## 1 Introduction

Primary biliary cholangitis (PBC) is a significant but relatively uncommon disease that primarily affects women. It is characterized as an autoimmune cholestatic liver disease, featuring distinct attributes such as cholestasis, serologic responsiveness to antimitochondrial antibodies (AMA) or antinuclear antibody (ANA) reactivity, combined, when it is necessary, with histological indications of chronic non-suppurative, granulomatous, lymphocytic small bile duct cholangitis (1–3). PBC is considered a rare disease, with a global incidence and prevalence estimated at 3 and 21.05 cases per 100,000, respectively. In Europe, the estimated incidence and prevalence are 2.57 and 25 cases per 100,000 (4). Similarly, Italy reported a point prevalence of 27.90 cases per 100,000 and an annual incidence of 5.3 cases per 100,000 inhabitants (5).

PBC significantly impacts patients in two main ways progression to end-stage liver disease (*e.g.*, cirrhosis, liver transplantation) and symptomatic manifestations.

These symptoms include cholestatic pruritus, sicca complex, cognitive symptoms, symptoms of social and emotional dysfunction, sleep disturbance and depression, abdominal discomfort and fatigue which may be impact on the patients’ quality of life (6, 7). However, the exact aetiology and pathogenesis of autoimmune diseases is not fully understood. Its pathogenesis likely involves both innate and adaptive immune responses, likely influenced by environmental factors interacting with immunogenetic and epigenetic backgrounds. This interaction leads to chronic immune-mediated biliary epithelial injury, causing cholestasis, ductopenia, hepatocyte injury, and eventual biliary fibrosis and cirrhosis (8–10).

Histologically, liver tissue in PBC shows damage to biliary epithelial cells (BECs) in relation to T cell, B cell, macrophage, eosinophil, and natural killer (NK) cell infiltration in the portal area (11). Another important aspect is the clear evidence of genetic susceptibility as indicated by family and twin studies. Among monozygotic twins, there is a 60% concordance rate, and the sibling relative risk is estimated to be 10.5 (12–15). In this context, the Human Leukocyte Antigen G (*HLA-G*) emerges as ideal candidate for delving into these pathologies. Specifically, this non-classical HLA-class I molecule actively contributes to the immunomodulatory functions mediated by NK cells (16, 17).

Indeed, it is a crucial component of the tolerogenic system (18). Influenced by genetic variability and environmental stimuli (19, 20), *HLA-G* expression is largely regulated by polymorphic sites in the 3’ untranslated region (*UTR*), impacting different plasma levels of HLA-G (sHLA-G) (21–24).

For instance, *HLA-G UTR-1* haplotype is known to produce the highest levels of soluble HLA-G (sHLA-G) (25). Globally, three main *HLA-G* alleles are associated with UTR-1: HLA-G*01:01:01:01/UTR-1 is the haplotype with the highest frequency (0.2277%), followed by haplotype HLA-G*01:01:01:08/UTR-1 (0.0204%), and the least frequent haplotype HLA-G*01:01:01:09/UTR-1 (0.0033%) (26).

Originally identified in placental cells, *HLA-G* expression extends to various non-fetal tissues (27, 28). Its immunosuppressive functions have been implicated in inflammation, infections, transplantation tolerance, and cancer where it has been demonstrated to facilitate immune evasion and tumor progression (18, 29–32). Notably, HLA-G’s involvement in liver-related conditions, such as chronic hepatitis B and C, underscored its role in liver homeostasis and injury response, with implications for fibrosis and hepatocellular carcinoma prognosis (33–37). Regarding autoimmune liver diseases, studies have demonstrated that type 1 autoimmune hepatitis (AIH-1) patients exhibit significantly lower levels of sHLA-G compared to the control group. Additionally, lower sHLA-G levels have been observed in AIH-1 patients with more severe disease (38). These findings are consistent with other autoimmune diseases like rheumatoid arthritis (38, 39), multiple sclerosis (40) and systemic lupus erythematosus (41). While *HLA* gene system has undergone comprehensive examination in the context of PBC (42), with in-depth investigations into polymorphisms conducted through large-scale studies (43). The findings from these studies indicate that susceptibility to PBC in white ethnic populations is associated with HLA DRB1*08 alleles. Conversely, a study focused on the Italian population indicates that the DRB1*11 allele has a protective effect against PBC (44).

However, the potential immunomodulatory role of *HLA-G* expression and its role in PBC, have not been investigated yet. In this context, Sardinia, being a genetic isolate, possesses homogeneous and lowly polymorphic genetic characteristics, leading to a higher prevalence of autoimmune diseases (e.g., type 1 diabetes, multiple sclerosis) compared to other populations (45, 46). This unique genetic landscape offers an opportunity for studying genetic associations in rare autoimmune diseases. The current research focuses on elucidating the function of HLA-G molecules in PBC, driven by their anti-inflammatory effects in autoimmune conditions. We investigate the role of *HLA-G* in PBC by examining both genetic variability and the phenotypic expression of soluble HLA-G and their impact on a group of PBC patients and their therapy, comparing it to a control group and another autoimmune liver disease (AIH-1) from the same geographic area (Sardinia, Italy).

## 2 Materials and methods

### 2.1 Patients and controls selection

A cohort of 166 Sardinian PBC patients was compared to a panel of 180 individuals from Sardinia, going back at least two generations of family history, has been utilized as healthy control population; they have been enrolled from the regional bone marrow registry to reflect the genetic background of Sardinian population (47). This population group accurately reflects the genetic profiles and male-to-female ratio of the population in the central-south geographical areas from where the PBC patients were recruited. The cohort of PBC patients was also compared to a panel of 205 AIH-1 patients originating from central-southern Sardinia who were referred to the same outpatient clinic. This comparison aimed to ensure the highest homogeneity in terms of genetic characteristics. The *HLA-G* alleles and *3’UTR* haplotypes of the patients and the healthy controls were compared. Patients’ and controls’ plasma sHLA-G levels were measured, and the results were categorized based on the several *HLA-G 3’UTR* haplotypes that have been linked to affecting HLA-G expression (30). Finally, sHLA-G levels were compared among the three extended haplotypes presenting the *HLA-G UTR-1* haplotype (HLA-G*01:01:01:01/UTR-1, HLA-G*01:01:01:08/UTR-1, and HLA-G*01:01:01:09/UTR-1).

The biochemical response to the therapy was assessed either using qualitative definitions based on discrete binary variables or through quantitative scoring systems computed from continuous parameters, as described in the EASL Clinical Practice Guideline (48).

In particular, at the Liver Unit of University Hospital of Cagliari, the criteria for selecting patients with an inadequate response involve those who, after 12 months of therapy, present ALP levels ≥ 1.5 x ULN (Paris-II criteria). sHLA-G levels were measured concurrently with the assessment of the biochemical response.

### 2.2 Ethics statement

Written informed consent was obtained from each patient or healthy subject included in the study, following the institutional and national ethical standards of the local human research committee. The study protocol, including informed consent procedures, conforms to the ethical guidelines of the Declaration of Helsinki and was approved by the responsible ethics committee (Ethics Committee of the Cagliari University Hospital; date of approval: January 23, 2014; protocol number NP/2014/456).

### 2.3 DNA extraction and HLA typing

Genomic DNA was extracted from peripheral blood mononuclear cells according to standard methods. Patients and controls were typed at high-resolution for the alleles at the *HLA-A, -B, -C -DR* loci and other 13 *HLA* loci (including *HLA-G*) using a Next-Generation Sequencing (NGS) method. AlloSeq Tx17 (CareDx). *HLA-G3’UTR* were sequenced using V3 flow cells for 300 cycle paired-end in a MiSeq platform (Illumina). The *HLA-G* gene region extending from positions −1550 to 3404, relative to the start codon, was analyzed using long-range PCR (49). The automatically generated FASTQ files were then processed with the MiSeq Reporter v2.6 for alignment and variant calling, and VariantStudio Software v3.0 for variant classification (Illumina, Netherlands).

### 2.4 Soluble HLA-G plasma quantification

Plasma samples were obtained from 166 PBC patients, 180 controls and 205 AIH patients at the time of enrolment. Levels of sHLA-G were determined by the sHLA- G ELISA assay kit (Exbio, Prague, Czech Republic) according to the manufacturer’s instructions. This kit detects both shedding HLA-G-1 and soluble HLA-G5 molecules. Briefly, plasma samples were immediately frozen after separation and stored at -80°C until use. Fifty μl of each sample were diluted 1:80 in the plasma-specific buffer prior to running the HLA-G assay. A six-point calibration curve was obtained using the human native HLA-G protein supplied with the kit. A microplate reader with a 450 nm filter was used to measure the optical density at the end of the reaction. The limit of sensitivity was 0.6 U/ml. All samples were assayed in duplicate.

### 2.5 Statistical analysis

Descriptive statistics for clinical and biochemical characteristics of PBC patients were reported as means with standard deviations (SD) for continuous variables, and as proportions for categorical variables. For all variables, 95% confidence intervals (CI) were provided. Group comparisons were conducted using the Student’s t-test for continuous data and Fisher’s exact test for categorical data, depending on the data distribution and sample size. Associations between *HLA-G* alleles or *HLA-G 3’UTR* haplotypes and different groups (PBC vs. healthy controls or AIH patients) were assessed using Fisher’s exact test. In particular, to correct for multiple comparisons involving *HLA-G 3’UTR* haplotypes, P-values were adjusted using the Bonferroni method, and only those with adjusted P-values (Pc) below 0.05 were considered as statistically significant.

Plasma concentrations of soluble HLA-G across PBC, AIH, and healthy control groups were summarized using means and 95% CIs. Moreover, violin plots were used to visualize the distribution of values, incorporating boxplots to display medians and interquartile ranges. Analyses also included stratification by UTR-1 haplotype status. Differences between groups were tested using the Student’s t-test, along with corresponding 95% CIs. All statistical analyses were carried out using R (version 4.3.2; R Core Team, 2023) (https://www.R-project.org/).

### 2.6 Patient selective algorithm

From 2014 to 2023 a cohort of 166 outpatients with PBC were enrolled from central-southern Sardinia who were referred to the Liver Unit of University Hospital of Cagliari. The diagnosis was made in accordance with EASL Clinical Practice Guidelines for the diagnosis and management of patients with primary biliary cholangitis (*e.g.*, elevation of ALP, AMA at a titre >1:40, specific ANA immunofluorescence, or histopathological features of PBC when biopsy is performed) (49).

**Figure 1:**
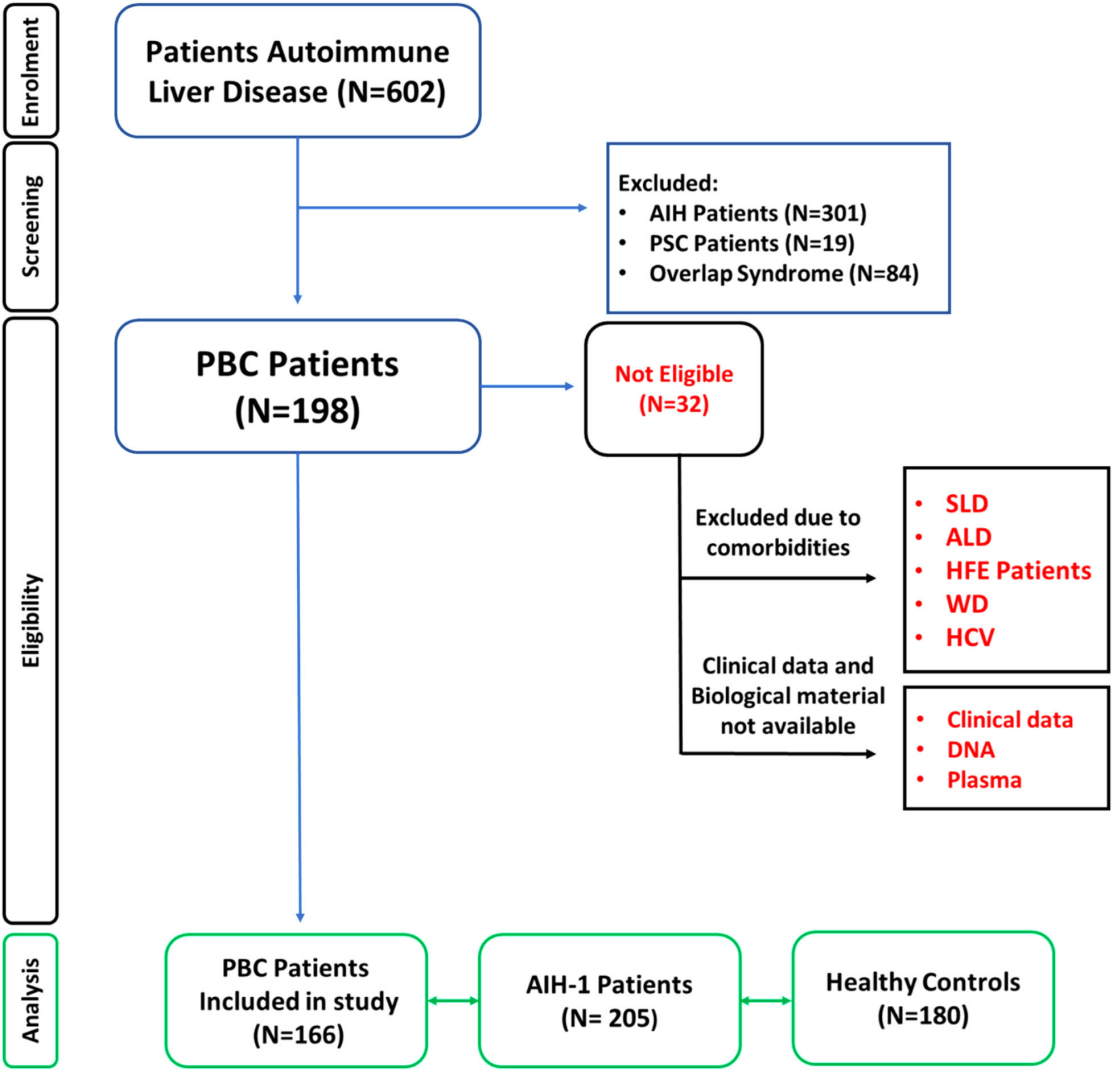
Patient enrolment workflow diagram. Overview of the study cohort selection according to EASL Clinical Practice Guidelines for the diagnosis and management of primary biliary cholangitis (PBC) (48). Patients with other chronic liver diseases were excluded. PBC, Primary biliary cholangitis; PSC, Primary sclerosing cholangitis; SLD, Steatosic liver disease; ALD, Alcoholic Fatty Liver Disease (50); HFE, Hereditary hemochromatosis; WD, Wilson Disease; HCV, Hepatitis C; HBV, Hepatitis B.

Patients with autoimmune hepatitis, overlap syndrome, and primary sclerosing cholangitis were excluded from the study to avoid bias. Additionally, individuals with chronic liver disorders caused by drug or alcohol abuse, fatty liver diseases, and metabolic or genetic disorders were excluded. Patients enrolled in the study tested negative for hepatitis B surface antigen (HbsAg), anti-hepatitis A virus IgM, anti-hepatitis C virus IgG antibody and anti-hepatitis D virus IgG antibody (**Figure 1**).

### 2.7 Clinical

The clinical, immunological and therapeutic characteristics of 166 PBC patients are shown in **Table 1**. The mean age at diagnosis was 55.8 ± 7.1 years and 147 patients (89%) were females. Most of the patients (74%) had high titres of anti-mitochondrial antibodies (AMA), detected either alone or in combination with antinuclear antibodies (ANA) in approximately half of the patients (51%). More than one-third of the patients (35%) presented one or more associated autoimmune diseases, including Hashimoto’s thyroiditis, Sjogren syndrome, type 1 diabetes, rheumatoid arthritis and other autoimmune diseases.

**Table 1:** Clinical, biochemical and therapeutic characteristics of PBC patients.

Characteristic	Total patients (166)	Non/partial responders (63)	Responders (103)	P value	OR (95% CI) or x_2_-x_1_^§^
Gender: n (%)					
Female	147 (0.886)	55 (0.873)	92 (0.893)	0.803	0.82 (0.28 - 2.51)
Male	19 (0.114)	8 (0.127)	11 (0.107)	0.803	1.22 (0.40 - 3.55)
Clinical characteristics, mean ± SD					
Age (yr)	67.8 ± 12.9	66.2 ± 5.7	68.8 ± 14.1	0.165	-2.6 (-6.3; 1.1)
Age at diagnosis (yr)	55.8 ± 7.1	52.9 ± 4.9	57.6 ± 8.2	**6.0·10^-5^**	-4.7 (-7.0; -2.4)
AST level (IU/L)	25.9 ± 8.7	29.8 ± 9.2	23.51 ± 7.8	**5.2·10^-6^**	6.3 (3.7; 8.9)
ALT level (IU/L)	25.8 ± 12	33.2 ± 14.1	21.3 ± 10.7	**5.7·10^-9^**	11.9 (8.1; 15.7)
GGT level (IU/L)*	61.0 ± 56.3	91.2 ± 33.9	42.5 ± 32.1	**1.2·10^-15^**	48.7 (38.3; 59.1)
ALP level (IU/L)^	115.9 ± 57	152.7 ± 21.2	93.4 ± 37.4	**< 2.2·10^-16^**	59.3 (49.1; 69.5)
Bilirubin (mg/dL)	2.5 ± 2.1	0.83 ± 0.3	3.52 ± 2.7	**4.6·10^-13^**	-2.69 (-3.36; -2.02)
Albumin (g/dL)	3.9 ± 0.4	3.87 ± 0.5	3.92 ± 0.3	0.421	-0.05 (-0.17; 0.07)
γ-globulin (g/dL)	1.01 ± 0.03	1.39 ± 0.08	0.77 ± 0.02	**< 2.2·10^-16^**	0.62 (0.60; 0.64)
PT-INR	1.2 ± 0.4	1.06 ± 0.12	1.29 ± 0.30	**3.3·10^-8^**	-0.23 (-0.31; -0.15)
Platelets (x10^3^/ul)	241 ± 83	239 ± 122	243 ± 95	0.814	-4 (-37; 29)
Autoimmune diseases, n (%)	58 (0.349)	24 (0.381)	34 (0.330)	0.508	1.25 (0.61 - 2.52)
Immunological characteristics n (%)					
ANA positivity	84 (0.506)	32 (0.508)	52 (0.505)	1	1.01 (0.52 - 1.99)
AMA positivity	123 (0.741)	44 (0.698)	79 (0.767)	0.364	0.71 (0.33 - 1.52)
sHLA-G U/mL, mean (95% CI)	9.10 ± 6.20	8.67 ± 7.11	13.81 ± 12.36	0.003	-5.14 (-8.51; -1.77)
Therapeutic characteristics, n (%)					
Ursodeoxycholic acid therapy	165 (0.994)	63 (100)	102 (0.990)	1	> 0.016
Obeticholic acid therapy	13 (0.078)	13 (0.206)	0 (0)	**1.4·10^-6^**	> 5.903

^§^Mean differences (for continuous variables): x_2_ (non-responders)-x_1_ (responders).

*^In our laboratory center the normal levels for GGT and ALP were in a range between 10–45 IU/L and 40–95 IU/L respectively, P-value calculated comparing responders vs non-responders.

ALP, Alkaline Phosphatase; ALT, Alanine Aminotransferase; AST, Aspartate Aminotransferase; GGT, Gamma-Glutamyl Transferase; PT-INR: Prothrombin Time – International Normalized Ratio; AMA, Antimitochondrial Antibodies; ANA, Antinuclear Antibodies; sHLA-G, Soluble Human Leukocyte Antigen G; OR, Odds Ratio; CI, Confidence Interval. Bold values indicate statistically significant results (P < 0.05 or Pc < 0.05).

At the latest clinical follow-up, the mean alkaline phosphatase (ALP) level was 115.9 ± 57 IU/L and the mean gamma glutamyl transferase (GGT) level was 61 ± 56.3 IU/L. In relation to cytolysis markers, the mean AST and ALT levels were 25.9 ± 8.7 IU/L and 25.8 ± 12 IU/L, respectively. Finally, the mean level of soluble HLA-G (sHLA-G) was 9.1 (3.5 – 22.3) U/mL (**Table 1**).

Subsequently, we assessed whether the presence of additional autoimmune conditions influenced sHLA-G levels in PBC patients. A subgroup analysis comparing patients with (n = 58) and without (n = 108) coexisting autoimmune diseases showed no statistically significant difference in sHLA-G concentrations [mean (95% CI): 10.14 (8.15 – 11.73) U/mL vs 8.14 (7.05 – 9.22) U/mL respectively; median (IQR): 8.08 (3.70 to 13.87) U/mL vs 6.28 (2.34 to 10.21) U/mL respectively; *P* = 0.117] (**Supplementary Figure S2**).

Finally, exploring the therapeutic approaches, nearly all of the patients (99%) had been treated with ursodeoxycholic acid, either alone or in combination with obeticholic acid (8%). One patient had discontinued UDCA therapy prior to sampling due to voluntary withdrawal. However, this individual showed no clinical flares or biochemical worsening during intermediate follow-up and maintained a stable disease course over 12 months. Therefore, the patient was included in the analysis and classified as a responder according to standard biochemical response criteria.

Instead, more than one-third of the patients (38%) were non-responders or partial responders to therapy. Among the laboratory markers, ALP and GGT were the most discordant parameters between the two patient groups [152.7 ± 21.2 IU/L vs 93.4 ± 37.4 IU/L; x_2_ - x_1_ = 59.3 (49.1; 69.5) IU/L; P< 2.2×10–^16^ and 91.2 ± 33.9 IU/L vs 42.5 ± 32.1 IU/L; x_2_ - x_1_ = 48.7 (38.3; 59.1) IU/L; P= 1.2×10^-15^, respectively] (**Table 1**).

Poor or partial responder patients exhibited clinical manifestations at a significantly younger age compared to the responder patient group 52.9 ± 4.9 yr vs 57.6 ± 8.2 yr respectively; x_2_ - x_1_ = -4.7 (-7.0; -2.4) yr; P = 6.0×10^-5^]. It is also interesting to observe that the sHLA-G levels in these patients were on average lower compared to those who responded positively to UDCA therapy [8.67 ± 7.11 U/mL vs 13.81 ± 12.36 U/mL respectively; x_2_ - x_1_ = -5.14 (-8.51; -1.77) U/mL; P = 0.003] (**Table 1**).

## 3 Results

### 3.1 Genetic analysis

#### 3.1.1 Comparison of HLA-G 3’UTR haplotype frequencies between PBC patients and controls

**Table 2** shows *3’UTR* haplotypes frequencies among 166 PBC patients and 180 healthy controls. The most prevalent haplotypes observed in both groups were: U*TR-1, UTR-2, UTR-5, UTR-3* and *UTR-7* [160/332 (48.2%), 36/332 (10.8%), 49/332 (14.8%), 35/332 (10.5%) and 32/332 (9.6%) in PBC patients vs 123/360 (34.3%), 92/360 (25.5%), 58/360 (15.9%), 30/360 (8.1%) and 24/360 (6.7%) in controls].

**Table 2:** Haplotype frequencies observed at the *HLA-G 3’ UTR* polymorphic sites (14bp Ins/Del, 3003C/T, 3010C/G, 3027A/C, 3035C/T, 3142C/G, 3187A/G, 3196C/G) in healthy controls and PBC patients.

HLA-G 3’UTR	180 Controls		166 PBC		Controls vs patients		
Haplotypes	2N=360	%	2N=332	%	P	OR (95% CI)	Pc
*UTR-1 (DelTGCCCGC)*	123	0.3429	160	0.482	**2.0−10^-4^**	1.792 (1.320 – 2.435)	**0.0018**
*UTR-2 (InsTCCCGAG)*	92	0.2548	36	0.108	**4.9−10^-7^**	0.354 (0.233 – 0.539)	**4.4−10^-6^**
*UTR-5 (InsTCCTGAC)*	58	0.1595	49	0.148	0.674	0.902 (0.596 – 1.363)	1
*UTR-3 (DelTCCCGAC)*	30	0.081	35	0.105	0.362	1.296 (0.777 – 2.164)	1
*UTR-7 (InsTCATGAC)*	24	0.0667	32	0.096	0.165	1.493 (0.860 – 2.593)	1
*UTR-4 (DelCGCCCAC)*	23	0.0619	18	0.054	0.631	0.840 (0.445 – 1.586)	1
*UTR-18 (DelTGCCCAC)*	4	0.0167	0	0	**0.031**	0 (0.000 – 0.914)	0.279
*UTR-10 (DelTCCCGAG)*	4	0.0119	1	0.003	0.375	0.269 (0.030 – 2.418)	1
*UTR-6 (DelTGCCCAC)*	2	0.0048	1	0.003	1	0.541 (0.049 – 5.992)	1

% = allele frequencies expressed as decimals. OR, Odds Ratio; CI, Confidence Interval. Bold values indicate statistically significant results (P < 0.05 or Pc < 0.05).

Interestingly, the *UTR-1* haplotype showed a significantly higher frequency in PBC patients than in controls [160/332 (48.2%) vs 123/360 (34.3%) respectively; OR = 1.79 (95% CI 1.32 – 2.44); P =2.0×10^-4^; Pc=0.0018].

On the other hand, the *UTR-2* haplotype was significantly less frequent in PBC patients than in control population [36/332 (10.8%) vs 92/360 (25.5%); OR = 0.35 (95% CI 0.23 – 0.54); P= 4.9×10^-7^; Pc= 4.4×10^-6^]. When comparing the frequencies of other *UTRs* between the PBC patient group and the control group, no statistical difference was observed.

#### 3.1.2 Comparison of HLA-G alleles frequencies between PBC patients and controls

The evaluation of *HLA-G* alleles and *3’UTR* haplotype frequencies between 166 PBC patients and 180 healthy controls has been detailed in **Table 3**. Analysis of the extended haplotypes *(HLA-G* alleles and *3’UTR* haplotypes) revealed some notable differences in frequencies between the patients and the healthy controls. The most frequent extended haplotypes in both groups were HLA-G*01:01:01:01/UTR-1, HLA-G*01:01:01:08/UTR-1, HLA-G*01:03:01:02/UTR-5, HLA-G*01:01:03:03/UTR-7 and HLA-G*01:01:02:01/UTR-2 [78/332 (23.5%), 77/332 (23.2%), 47/332 (14.2%), 32/332 (9.6%) and 18/332 (5.4%) in PBC patients vs 77/360 (21.4%), 45/360 (12.5%), 55/360 (15.3%), 24/360 (6.7%) and 43/360 (11.9%) in controls]. The most relevant difference was observed in the frequencies of extended haplotype HLA-G*01:01:01:08/UTR-1 that was significantly more present in PBC patients than in controls [77/332 (23.2%) vs 45/360 (12.5%); OR = 2.11 (95% CI 1.41 – 3.16); P= 3.0×10^-4^; P_c_=0.008]. In contrast, the extended haplotype HLA-G*01:01:02:01/UTR-2 exhibited a significantly lower frequency in patients compared to the controls; however, this association did not remain statistically significant after correction for multiple testing [18/332 (5.4%) vs 43/360 (11.9%); OR = 0.42 (95% CI 0.24 – 0.75); P = 0.003; P_c_=0.075]. The prevalence of the extended haplotype HLA-G*01:03:01:02/UTR-5 was approximately similar in PBC patients and controls [47/332 (14.2%) vs 55/360 (15.3%); OR = 0.92 (95% CI 0.60 – 1.39); P = 0.748; P_c_= 1.00]. The occurrences of additional extended haplotypes were below 3%, giving a statistical assessment of the disparities between the two groups not achievable.

**Table 3:** Extended haplotypes (*HLA-G* alleles and *3’UTR* haplotypes) frequencies in population controls and PBC patients.

Extended haplotypes		180 Controls		166 PBC patients		Controls vs Patients		
Alleles	3’UTR haplotypes	2N = 360	*%*	2N = 332	*%*	*P* value	OR (95% CI)	*P_c_*
G*01:01:01:01	UTR-1	77	0.214	78	0.235	0.524	1.129 (0.789 – 1.614)	1
G*01:03:01:02	UTR-5	55	0.153	47	0.142	0.748	0.915 (0.600 – 1.394)	1
G*01:01:02:01	UTR-2	43	0.119	18	0.054	**0.003**	0.423 (0.239 – 0.749)	0.075
G*01:01:03:03	UTR-7	24	0.067	32	0.096	0.165	1.493 (0.860 – 2.593)	1
G*01:01:01:08	UTR-1	45	0.125	77	0.232	**3.0−10^-4^**	2.114 (1.412 – 3.163)	**0.008**
G*01:01:22:01	UTR-2	22	0.061	8	0.024	**0.024**	0.279 (0.167 – 0.864)	0.600
G*01:04:01:01	UTR-3	20	0.056	24	0.072	0.436	1.325 (0.718 – 2.446)	1
G*01:01:01:05	UTR-4	18	0.050	16	0.048	1	0.962 (0.482 – 1.919)	1
G*01:06:01:01	UTR-2	13	0.036	3	0.009	**0.021**	0.243 (0.069 – 0.862)	0.525
G*01:05N	UTR-2	7	0.019	4	0.012	0.549	0.615 (0.178 – 2.120)	1
G*01:04:04	UTR-3	6	0.017	11	0.033	0.219	2.022 (0.739 – 5.530)	1
G*01:01:01:04	UTR-18	4	0.011	0	0.000	0.125	0 (0.000 – 1.638)	1
G*01:04:01:01	UTR-10	4	0.011	1	0.003	0.375	0.269 (0.030 – 2.418)	1
G*01:06:01:02	UTR-2	3	0.008	1	0.003	0.625	0.360 (0.037 – 3.473)	1
G*01:01:01:06	UTR-4	3	0.008	1	0.003	0.625	0.360 (0.037 – 3.473)	1
G*01:04:01:02	UTR-3	3	0.008	0	0.000	0.250	0 (0.000 – 2.620)	1
G*01:03:01:01	UTR-5	3	0.008	2	0.006	1	0.721 (0.120 – 4.343)	1
G*01:01:01:01	UTR-4	2	0.006	1	0.003	1	0.541 (0.049 – 5.992)	1
G*01:01:01:04	UTR-6	2	0.006	1	0.003	1	0.541 (0.049 – 5.992)	1
G*01:01:01:06	UTR-2	2	0.006	1	0.003	1	0.541 (0.049 – 5.992)	1
G*01:06:02:02	UTR-2	1	0.003	0	0.000	1	0 (0.000 – 42.290)	1
G*01:01:01:01	UTR-3	1	0.003	0	0.000	1	0 (0.000 – 42.290)	1
G*01:02:02	UTR-2	1	0.003	0	0.000	1	0 (0.000 – 42.290)	1
G*01:01:01:09	UTR-1	1	0.003	5	0.015	0.110	5.489 (0.638 – 47.231)	1
G*01:01:01:08	UTR-2	0	0.000	1	0.003	0.480	> 0.028	1

% = allele frequencies expressed as decimals. OR, Odds Ratio; CI, Confidence Interval. Bold values indicate statistically significant results (P < 0.05 or Pc < 0.05).

#### 3.1.3 Comparison of HLA-G 3’UTR haplotype frequencies between PBC and AIH patients

The *UTR-1* haplotype was the most prevalent among both PBC and AIH patients, with frequencies of 48.2% (160 out of 332) and 40.2% (165 out of 410) respectively (**Table 4**). Although a statistical significance was not reached [OR = 1.38 (95% CI 1.03 – 1.85); P=0.031; Pc=0.248]. Similarly, *UTR-5* haplotype showed a higher prevalence in PBC patients compared to those with AIH (14.8% (49/332) and 8.5% (35/410) respectively). However, once more, no significant difference was observed between the two groups [OR = 1.86 (95% CI 1.17 – 2.94); P=0.010; Pc = 0.080]. Conversely, the *UTR-2* haplotype is significantly less represented in PBC patients compared to those with AIH [10.8% (36/332) and 25.6% (105/410) respectively, OR = 0.35 (95% CI 0.23 – 0.53); P = 2.9 x 10^-7^; Pc = 2.3×10^-6^].

**Table 4:** Haplotype frequencies observed at the *HLA-G 3’UTR* polymorphic sites (14bp Ins/Del, 3003C/T, 3010CG, 3027A/C, 3035C/T, 3142C/G, 3187A/G, 3196C/G) in PBC and AIH type 1 patients.

HLA-G 3’UTR haplotypes	166 PBC patients 2N=332		205 AIH patients 2N=410		PBC Patients vs AIH Patients		
	*2N*	*%*	*2N*	*%*	*P*	OR (95% CI)	*P_c_*
*UTR-1 (DelTGCCCGC)*	160	0.482	165	0.402	**0.031**	1.381 (1.031 – 1.850)	0.248
*UTR-5 (InsTCCTGAC)*	49	0.148	35	0.085	**0.010**	1.855 (1.171 – 2.940)	0.080
*UTR-2 (InsTCCCGAG)*	36	0.108	105	0.256	**2.9−10^-7^**	0.353 (0.234 – 0.533)	**2.3−10^-6^**
*UTR-3 (DelTCCCGAC)*	35	0.105	30	0.073	0.150	1.493 (0.896 – 2.488)	1
*UTR-7 (InsTCATGAC)*	32	0.096	25	0.061	0.095	1.643 (0.953 – 2.832)	0.760
*UTR-4 (DelCGCCCAC)*	18	0.054	35	0.085	0.115	0.614 (0.341 – 1.106)	0.920
*UTR-10 (DelTCCCGAG)*	1	0.003	5	0.012	0.232	0.245 (0.028 – 2.105)	1
*UTR-6 (DelTGCCCAC)*	1	0.003	10	0.024	**0.028**	0.121 (0.015 – 0.949)	0.224

2N *=* number of HLA-G *3’UTR* haplotypes. % = allele frequencies expressed as decimals. OR, Odds Ratio; CI, Confidence Interval. Bold values indicate statistically significant results (P < 0.05 or Pc < 0.05).

#### 3.1.4 Comparison of HLA-G alleles frequencies between PBC and AIH patients

**Table 5** outlines the comparison of *HLA-G* alleles and *3’UTR* haplotype frequencies between 166 PBC patients and 205 AIH patients. In this comparison as well, the most significant difference in frequencies was observed in the extended haplotype HLA-G*01:01:01:08/UTR-1, which exhibited a notably higher presence in PBC patients compared to AIH patients [77/332 (23.2%) vs 27/410 (6.6%); OR=4.28 (95% CI 2.64 – 7.10); P= 1.2×10^-10^; P_c_=2.6×10^-9^]. In contrast, HLA-G*01:01:01:01/UTR-1 was statistically significantly less present in PBC than in AIH patients [78/332 (23.5%) vs 138/410 (33.7%); OR=0.61 (95% CI 0.44 – 0.84); P=0.003]; however, the association did not remain statistically significant after correction for multiple testing (Pc = 0.066). Among the most frequent extended haplotypes (>3%), stands out the low frequency of HLA-G*01:01:02:01/UTR-2 in PBC compared to those in AIH-1 patients [18/332 (5.4%) vs 65/410 (15.9%); OR = 0.30 (95% CI 0.18 – 0.52); P= 6.1×10^-6^; P_c_= 1.3×10^-4^]. Another interesting observation is the increased frequency of two extended haplotypes, HLA-G*01:03:01:02/UTR-5 and HLA-G*01:01:03:03/UTR-7, in PBC patients compared to AIH-1 patients. However, only HLA-G*01:03:01:02/UTR-5 showed a statistically significant difference at the nominal level [47/332 (14.2%) vs 35/410 (8.5%); OR = 1.77 (95% CI 1.11–2.81); P = 0.018], which did not remain significant after correction for multiple testing (Pc = 0.396). The HLA-G*01:01:03:03/UTR-7 haplotype, also showed a higher frequency in PBC patients, though without reaching statistical significance [32/332 (9.6%) vs 25/410 (6.1%); OR = 1.64 (95% CI 0.95–2.83); P = 0.095; Pc = 1.00] Frequencies of other extended haplotypes were less than 3%, showing no significant differences between the two groups (PBC and AIH-1 patients).

**Table 5:** Extended haplotypes (HLA-G alleles and 3’UTR haplotypes) frequencies in PBC and AIH type 1 patients.

Extended haplotypes		166 PBC patients, 2N = 332		205 AIH patients, 2N = 410		PBC Patients vs AIH Patients		
Alleles	3’UTR haplotypes	*2N*	*f (%)*	*2N*	*f (%)*	*P* value	OR* (95% CI)	*P_c_*
HLA-G*01:01:01:01	*UTR-1*	78	0.235	138	0.337	**0.003**	0.605 (0.437 – 0.839)	0.066
HLA-G*01:01:01:08	*UTR-1*	77	0.232	27	0.066	**1.2−10^-10^**	4.275 (2.641 – 7.098)	**2.6−10^-9^**
HLA-G*01:03:01:02	*UTR-5*	47	0.142	35	0.085	**0.018**	1.767 (1.111 – 2.810)	0.396
HLA-G*01:01:03:03	*UTR-7*	32	0.096	25	0.061	0.095	1.643 (0.953 – 2.832)	1
HLA-G*01:04:01:01	*UTR-3*	24	0.072	26	0.063	0.661	1.151 (0.648 – 2.045)	1
HLA-G*01:01:02:01	*UTR-2*	18	0.054	65	0.159	**6.1−10^-6^**	0.304 (0.177 – 0.524)	**1.3−10^-4^**
HLA-G*01:01:01:05	*UTR-4*	16	0.048	35	0.085	0.057	0.542 (0.295 – 0.999)	1
HLA-G*01:04:04	*UTR-3*	11	0.033	4	0.010	**0.034**	3.478 (1.097 – 11.025)	0.748
HLA-G*01:01:22:01	*UTR-2*	8	0.024	5	0.012	0.266	2.000 (0.648 – 6.172)	1
HLA-G*01:01:01:09	*UTR-1*	5	0.015	0	0.000	**0.018**	> 1.139	0.396
HLA-G*01:05N	*UTR-2*	4	0.012	10	0.024	0.282	0.488 (0.152 – 1.570)	1
HLA-G*01:06:01:01	*UTR-2*	3	0.009	15	0.037	**0.016**	0.240 (0.069 – 0.837)	0.352
HLA-G*01:03:01:01	*UTR-5*	2	0.006	0	0.000	0.200	> 0.232	1
HLA-G*01:04:01:01	*UTR-10*	1	0.003	2	0.005	1	0.616 (0.056 – 6.872)	1
HLA-G*01:06:01:02	*UTR-2*	1	0.003	3	0.007	0.632	0.410 (0.042 – 3.959)	1
HLA-G*01:01:01:06	*UTR-4*	1	0.003	0	0.000	0.447	> 0.032	1
HLA-G*01:01:01:01	*UTR-4*	1	0.003	0	0.000	0.447	> 0.032	1
HLA-G*01:01:01:04	*UTR-6*	1	0.003	10	0.024	**0.028**	0.121 (0.015 – 0.949)	0.616
HLA-G*01:01:01:06	*UTR-2*	1	0.003	1	0.002	1	1.236 (0.077 – 19.830)	1
HLA-G*01:06:02:02	*UTR-2*	1	0.000	2	0.005	0.505	0 (0.000 – 6.575)	1
HLA-G*01:04:01:01	*UTR-2*	0	0.000	4	0.010	0.132	0 (0.000 – 1.866)	1
HLA-G*01:01:22:01	*UTR-10*	0	0.000	3	0.007	0.257	0 (0.000 – 2.986)	1

2N = number of extended haplotypes. % = allele frequencies expressed as decimals. OR, Odds Ratio; CI, Confidence Interval. Bold values indicate statistically significant results (P < 0.05 or Pc < 0.05).

### 3.2 Soluble HLA-G dosage

#### 3.2.1 Comparison sHLA-G between PBC and population control group

**Figure 2:**
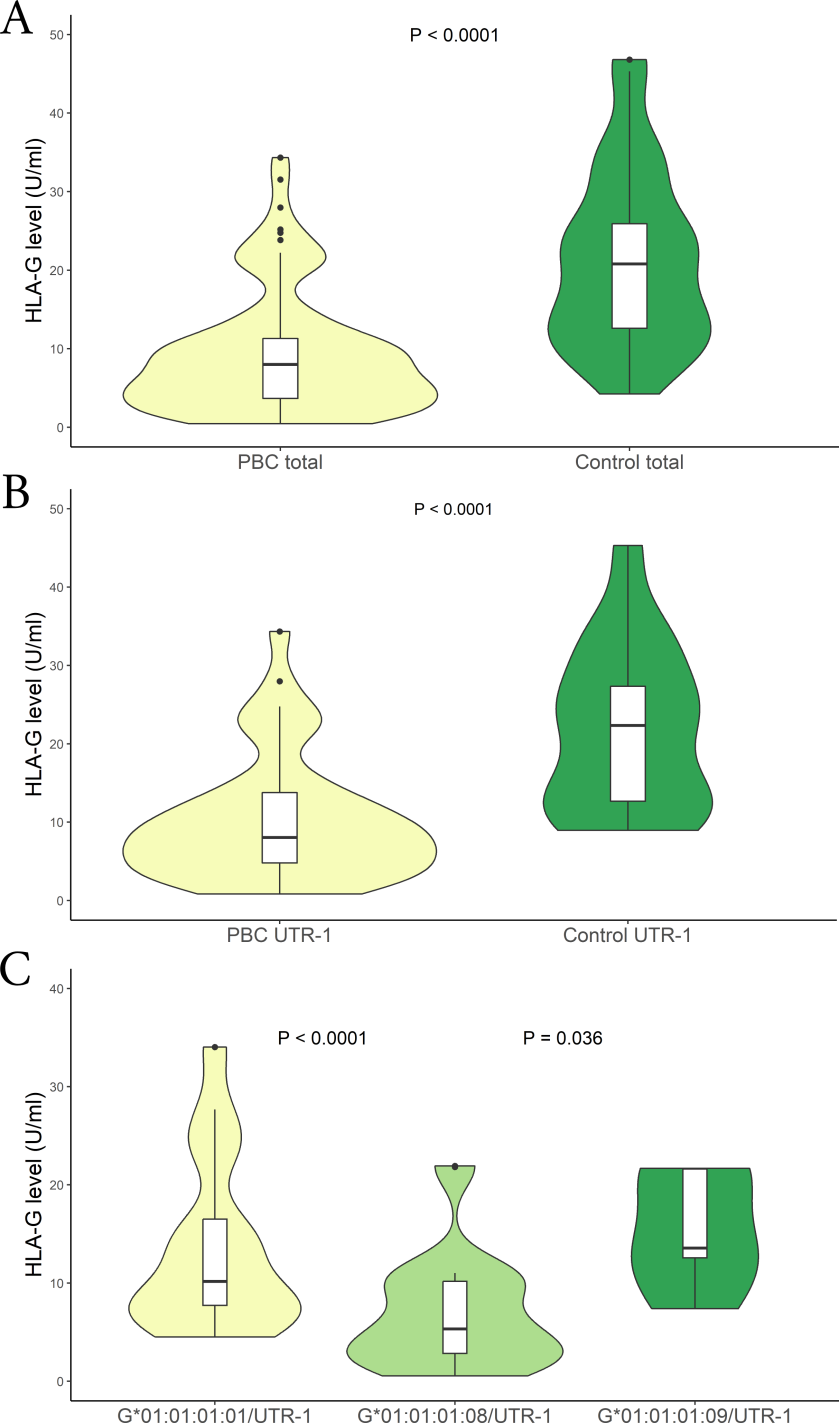
**(A)** Soluble HLA-G plasma levels (U/mL) were compared between in PBC patients (yellow) and control population (green). The P values reported was computed using the Student’s t test. The boxplot is included in the violin to assess the median and interquartile range. **(B)** Soluble HLA-G plasma levels (U/mL) were compared between in PBC patients (yellow) and controls who carried the HLA-G UTR-1 haplotype (green). The P values reported was computed using the Student’s t test. The boxplot is included in the violin to assess the median and interquartile range. **(C)** Soluble HLA-G plasma levels (U/mL) were compared between in PBC patients who carried the HLA-G*01:01:01:08/UTR-1 (light-green), G*01:01:01:01/UTR-1 (yellow) and G*01:01:01:09/UTR-1 (green) alleles combined with UTR-1. The P values reported was computed using the Student’s t test. The boxplot is included in the violin to assess the median and interquartile range.

Soluble HLA-G (sHLA-G) levels were measured in patients and healthy controls at the time of enrolment. The levels of sHLA-G were significantly lower in PBC patients than in controls [mean (95% CI): 9.10 (8.15 – 10.05) U/mL vs 24.03 (19.08 – 28.98) U/mL respectively; P < 0.0001; median (IQR): 7.98 (3.73 to 11.24) U/mL vs 20.73 (12.64 to 25.80) U/mL respectively] (**Figure 2A**). In addition, both patients and controls were divided according to the *HLA-G 3’UTR* haplotype. The only significant finding relates to the *UTR-1* haplotype. Specifically, the expression of sHLA-G was significantly lower in PBC patients compared to controls [mean (95% CI): 10.20 (8.85 – 11.55) U/mL vs 26.19 (19.00 – 33.38) U/mL respectively; P < 0.0001; median (IQR): 8.05 (4.81 to 13.82) U/mL vs 22.46 (12.78 to 27.54) U/mL respectively] *(* **Figure 2B**). To further explore these findings, we considered *HLA-G* alleles which are in linkage disequilibrium within the extended haplotype containing *UTR-1* (HLA-G*01:01:01:08, HLA-G*01:01:01:01 and HLA-G*01:01:01:09) (26). The analysis indicated that patients carrying the HLA-G*01:01:01:08 allele exhibited significantly lower levels of sHLA-G [mean (95% CI): 6.92 (5.57 – 8.27) U/mL; median (IQR): 5.29 (2.79 to 10.12) U/mL] compared to those with the HLA-G*01:01:01:01 [mean (95% CI): 13.50 (11.10 – 15.89) U/mL; median (IQR): 10.13 (7.75 to 16.58) U/mL] and HLA-G*01:01:01:09 [mean (95% CI): 15.36 (7.67 – 23.05) U/mL; median (IQR): 13.58 (12.58 to 21.58) U/mL] alleles [P < 0.0001 and P = 0.036 respectively] (**Figure 2C**).

#### 3.2.2 Comparison sHLA-G between PBC and AIH-1 patients

**Figure 3:**
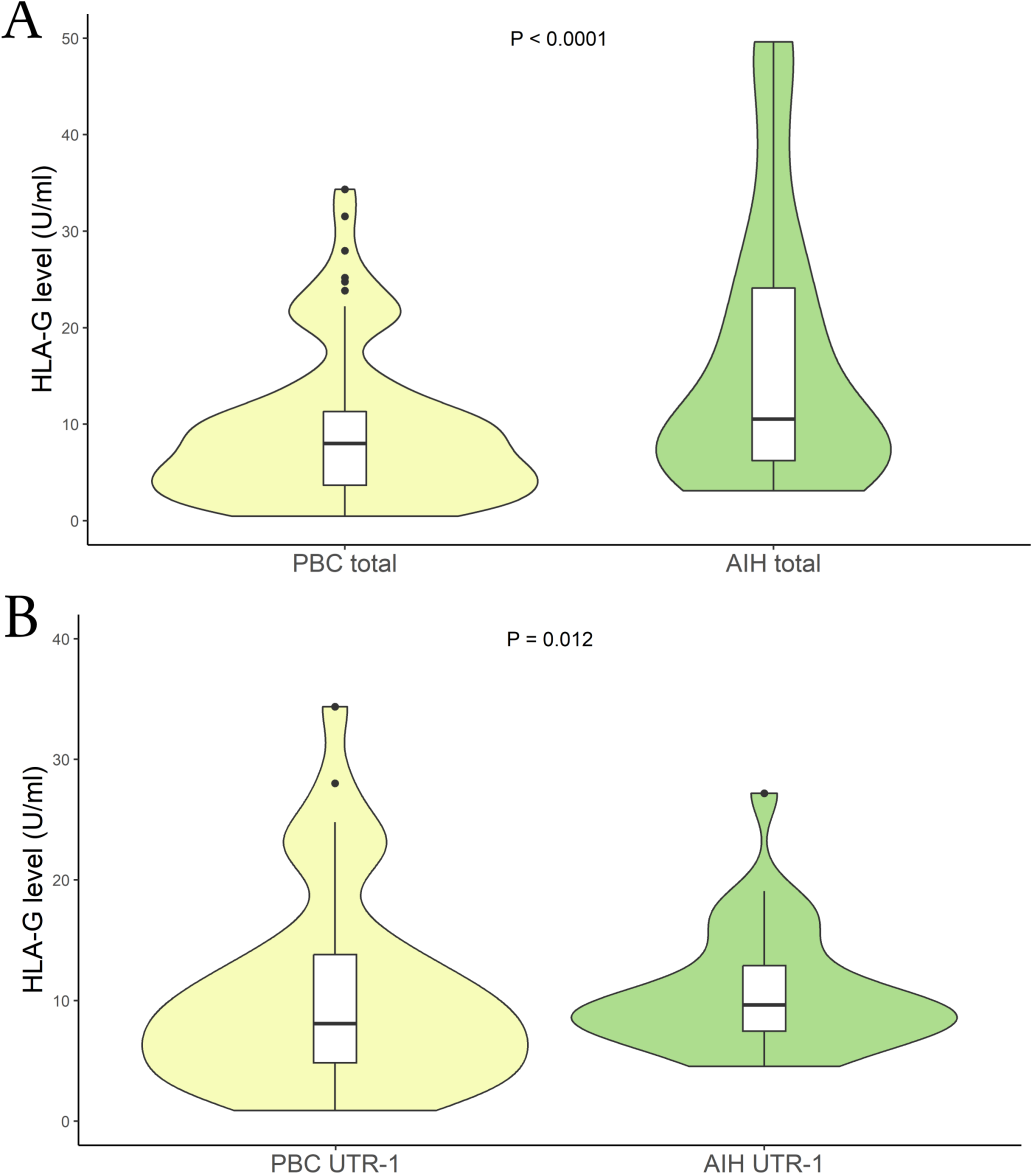
**(A)** Soluble HLA-G plasma levels (U/mL) were compared between in PBC (yellow) and AIH patients (light-green). The P values reported was computed using the Student’s t test. The boxplot is included in the violin to assess the median and interquartile range. **(B)** Soluble HLA-G plasma levels (U/mL) were compared between PBC (yellow) and AIH patients who carried the HLA-G UTR-1 haplotype (light-green). The P values reported was computed using the Student’s t test. The boxplot is included in the violin to assess the median and interquartile range.

We then aimed to verify if there were differences in the sHLA-G levels among patients with PBC and AIH. Comparing those two groups of patients we found significantly lower sHLA-G levels in PBC than in AIH patients [mean (95% CI): 9.10 (8.15 – 10.05) U/mL vs 13.9 (11.6 - 17.4) U/mL respectively; P < 0.0001; median (IQR): 7.98 (3.73 to 11.24) U/mL vs 10.52 (6.32 to 24.09) U/mL respectively] (**Figure 3A**). Following the previous analysis, we categorized the patients based on their HLA-G *3’UTR* haplotypes. Once again, the only significant difference observed was associated with the *UTR-1* haplotype. Specifically, within the PBC patient group, lower levels of sHLA-G were found compared to the AIH patients [mean (95% CI): 10.20 (8.85 – 11.55) U/mL vs 11.2 (9.0 – 14.8) U/mL respectively; P = 0.012; median (IQR): 8.05 (4.81 to 13.82) U/mL vs 9.63 (7.47 to 12.86) U/mL respectively] (**Figure 3B**). Moreover, comparing PBC and AIH patients that do not carry UTR-1, no significant difference in HLA-G levels was found.

#### 3.2.3 Soluble HLA-G dosage and therapy response

**Figure 4:**
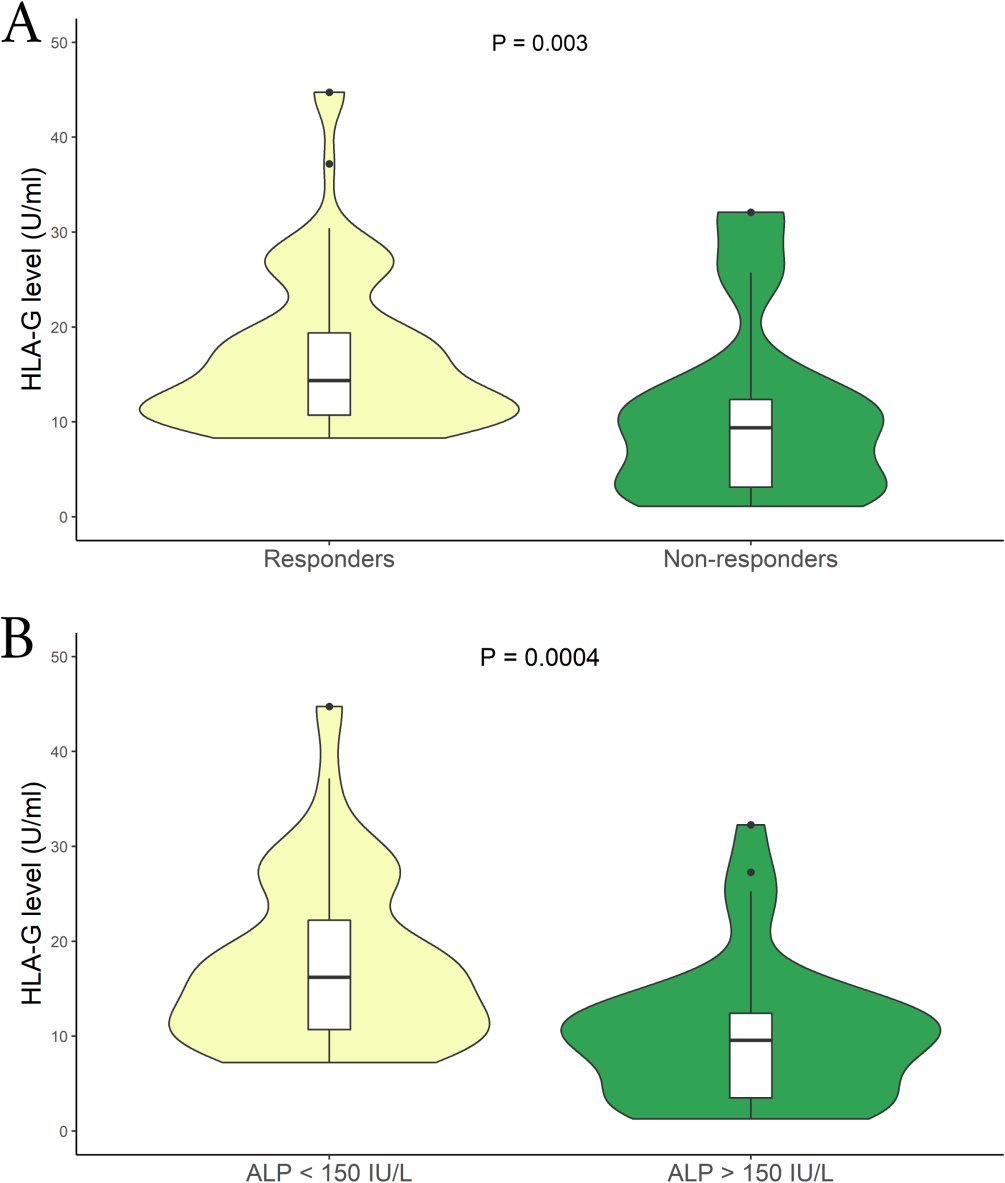
**(A)** Soluble HLA-G plasma levels (U/mL) were compared between Responder (yellow) and Non-responders patients in relation to the therapy (green). The P values reported was computed using the Student’s t test. The boxplot is included in the violin to assess the median and interquartile range. **(B)** Comparison of soluble HLA-G plasma levels (U/mL) among patients relative to their ALP (IU/L) levels; ALP < 1.5 x ULN (yellow) and ALP > 1.5 x ULN (green). The P values reported was computed using the Student’s t test. The boxplot is included in the violin to assess the median and interquartile range.

Another crucial aspect we aimed to explore is the correlation between sHLA-G levels and patients’ response to therapy. We divided the patients into two groups based on their therapy response status (responder vs. non-responder), following the criteria described above. The analysis revealed a substantial difference between the two patient groups. Specifically, among PBC patients with an inadequate therapy response, we observed lower sHLA-G levels compared to those who responded positively to the UDCA therapy [mean (95% CI): 8.67 (6.50 – 10.08) U/mL vs 13.81 (10.65 – 15.48) U/mL respectively; P = 0.003; median (IQR): 9.38 (3.16 to 12.33) U/mL vs 14.35 (10.78 to 19.38) U/mL respectively] (**Figure 4A**). Moreover, to support these findings, we considered patients with ALP levels >1.5 x ULN as indicative of an altered status (no responders). **Figure 4B** illustrates a significant difference between the two groups. Patients with ALP values above the threshold exhibited lower sHLA-G levels [mean (95% CI): 7.26 (2.7 – 29.1) U/mL vs 15.01 (6.53 – 59.53) U/mL respectively; P = 0.0004; median (IQR): 9.58 (3.54 to 12.39) U/mL vs 16.25 (10.78 to 22.29) U/mL respectively]. Notably, these sHLA-G levels, alongside those observed in patients carrying the HLA-G*01:01:01:08/UTR-1 extended haplotype, were among the lowest measured in our entire cohort (**Supplementary Figure S1**).

Finally, we sought to examine the presence of the HLA-G*01:01:01:08/UTR-1 extended haplotype concerning therapy response and ALP levels. As summarized in **Table 6**, the presence of this allele is notably higher in patients with a worse therapy response [60% vs 24.1% respectively; P = 0.0001], as well as in patients with ALP levels > 1.5 x ULN [51.3% vs 30.1% respectively; P=0.029].

**Table 6:** Patients stratified after 1 year of UDCA therapy, based on response and serological parameters ALP and GGT.

Clinical characteristics	Categories	G*01:01:01:08/UTR-1 presence (%)	P value	OR (95% CI)
Therapy response	No Responders	60.00	**1.0−10^-4^**	4.65 (2.03 – 10.98)
	Responders	24.14		
ALP	ALP < 1.5 x ULN	30.11	**0.029**	0.41 (0.18 – 0.95)
	ALP > 1.5 x ULN	51.28		
GGT	GGT < 1 x ULN	33.33	0.334	0.67 (0.30 – 1.52)
	GGT > 1 x ULN	42.86		

ALP, Alkaline Phosphatase; GGT, Gamma-Glutamyl Transferase; OR, Odds Ratio; CI, Confidence Interval. Bold values indicate statistically significant results (P < 0.05).

## 4 Discussion

The Sardinian population and its genetic makeup provide a valuable research opportunity for investigating complex diseases such as primary biliary cholangitis (PBC) (51). This genetic background enables significant insights even with small sample sizes, particularly in rare autoimmune diseases. Although several studies have examined the role of HLA-G in autoimmunity (24, 38–40), no previous work has specifically investigated the genetic and molecular profile of HLA-G in PBC. Previous GWAS studies have suggested a potential correlation between HLA-G and PBC, highlighting the involvement of immune system pathways in the disease’s pathogenesis (52).

Our study is the first to identify a potential correlation between HLA-G genetic variability, sHLA-G levels, and PBC pathogenesis. We found that the *UTR-1* haplotype, which is known to stabilize mRNA and produce higher levels of sHLA-G (33), was significantly more frequent in PBC patients compared to controls (48.2% vs 34.3%). However, this frequency was not significantly different between PBC and AIH-1 patients.

Despite the expected association between *UTR-1* and higher sHLA-G levels, PBC patients showed significantly lower sHLA-G concentrations than both controls and AIH-1 patients (9.1 vs 24.03 vs 13.9 U/mL, respectively). This discrepancy was especially pronounced in PBC patients carrying the *UTR-1* haplotype, suggesting that disease-specific mechanisms may override the expected genetic influence on sHLA-G expression.

HLA-G exists in both membrane-bound and soluble forms. Soluble isoforms (HLA-G5 to -G7) arise from alternative splicing, while HLA-G1 can be cleaved into soluble form by metalloproteases (MMPs) such as MMP-2 (53, 54). Both isoforms play immunomodulatory roles. This lower level of sHLA-G, particularly in comparison to healthy populations, is consistent with findings in other autoimmune diseases such as systemic sclerosis (SSc), rheumatoid arthritis, juvenile idiopathic arthritis, and systemic lupus erythematosus, suggest that low sHLA-G expression correlates with high disease activity (55). For instance, a recent study by Favoino et al. reported that patients with lower sHLA-G levels experienced more severe SSc disease (55).

The causes behind the low sHLA-G levels in PBC remain unclear. One possibility is reduced cleavage of membrane-bound HLA-G1 by MMP. Dysregulation of MMP activity in cholestatic liver injury have been discovered; experimental models of bile duct ligation show that MMP-2 and MMP-9 activity increases early and remains elevated (56). Nonetheless, higher MMP activity would predict increased sHLA-G levels, which we did not observe. A possible explanation is the presence of functional polymorphisms in MMP genes that affect their activity (57), potentially modulating sHLA-G shedding at an individual level.

Another involves post-transcriptional regulation, including the influence of miRNAs such as *miR-148a* known to downregulate sHLA-G in the placenta of patients with intrahepatic cholestasis of pregnancy (ICP) and negatively correlates with serum total bile acid levels (58). Given that PBC shares immunological and cholestatic features with ICP, it is plausible that similar miRNA-mediated downregulation could contribute to the unexpectedly low sHLA-G levels observed in our PBC cohort, despite the presence of the *UTR-1* haplotype associated with higher HLA-G expression.

Moreover, the role of membrane-bound HLA-G in the liver microenvironment also warrants attention. In AIH-1 and HCV-infected livers, hepatic HLA-G expressions have been linked to histological severity and fibrosis (36, 38) possibly reflecting a mechanism of immune modulation or cellular self-protection. Although immunohistochemical data on HLA-G expression in liver tissue are currently lacking for PBC, it is plausible that individual variability in membranous HLA-G expression among hepatic immune cells could contribute to the differences in sHLA-G levels observed in plasma. Furthermore, the reduced presence of sHLA-G may reflect impaired proteolytic shedding, potentially resulting in local retention of membrane-bound HLA-G. Such retention could enhance localized immunomodulatory activity, possibly promoting an environment of sustained immune dysregulation and contributing to the increased histopathological severity observed in affected liver tissue.

Notably, in Sardinian population, the *HLA-G UTR-1* haplotype is associated with three *HLA-G* alleles: HLA-G*01:01:01:01, HLA-G*01:01:01:09 and HLA-G*01:01:01:08, which was significantly more frequent in PBC patients compared to controls and AIH patients (0.232% vs 0.125% vs 0.066%). This allele, combined with *UTR-1*, was associated with significantly lower sHLA-G levels compared to other *UTR-1* haplotypes (HLA-G*01:01:01:01/UTR-1 and HLA-G*01:01:01:09/UTR-1; P < 0.0001 and P = 0.036 respectively) (**Figure 2C**).

Despite encoding the full-length, functional HLA-G1 isoform, its lower soluble levels suggest that specific extended haplotypes may influence HLA-G regulation through mechanisms that extend beyond simple transcript abundance, potentially affecting post-transcriptional processes such as membrane expression dynamics or proteolytic cleavage efficiency.

The combination of high frequency and low sHLA-G levels in HLA-G*01:01:01:08/UTR-1 carriers support its role as a genetic risk factor for PBC. Intriguingly, this haplotype was also associated with poorer response to UDCA therapy. Patients who had a good response to 12 months of UDCA therapy exhibited significantly higher sHLA-G levels compared to those with inadequate responses. Elevated alkaline phosphatase (ALP) levels (> 1.5 x ULN), which indicate poorly controlled disease or progression, were inversely proportional to sHLA-G levels. Other studies have identified clear differences in both “immune” and “senescence” phenotypes between low-risk and high-risk PBC patients from the early stages of the disease. The low-risk disease appears to be characterized by cholestatic bile damage without any immune-mediated injury. Indeed, these low-risk PBC patients are well responsive to the detoxifying effects of UDCA (59). In contrast, high-risk disease is characterized by T-cell activation and apoptosis, leading to continuous bile duct damage which is not treatable by UDCA alone (59).

This distinction may relate to our findings; indeed, the HLA-G*01:01:01:08/UTR-1 extended haplotype could help identify patients with poorer therapy responses, associated with different immune phenotypes exhibiting enhanced T-cell activity (60% vs 24.1%; P < 1.0×10^-4^). It is well-known that HLA-G molecules are involved in the inhibition of NK cell activity, CD4+ T lymphocyte responses, and dendritic cell maturation, as well as in the apoptosis of CD8+ cytotoxic T cells and the development of regulatory T cells (60).

In PBC, autoreactive CD4^+^ and CD8^+^ T cells contribute to bile duct damage via recognition of mitochondrial antigens such as the E2 subunit of the pyruvate dehydrogenase complex (61). Thus, reduced sHLA-G may fail to control autoreactive T cells, perpetuating inflammation. Indeed, HLA-G has been shown to induce apoptosis in CD8^+^ T cells and to reduce the proliferation of CD4^+^ T cells and B cells (62).

Our data suggest that HLA-G*01:01:01:08/UTR-1 may therefore support the idea that it could dysregulate the inflammatory and immune response, resulting in an increased susceptibility to the disease and its severity. Currently, no therapies specifically target HLA-G for the treatment of autoimmune and autoinflammatory diseases. However, research in experimental models, such as uveitis, has shown that increasing HLA-G levels can significantly improve clinical manifestations, indicating its potential as a therapeutic target (63).

An additional consideration is the presence of other autoimmune diseases in our cohort. PBC can frequently coexist with concomitant extrahepatic autoimmune diseases (64), potentially confounding immunological analyses and limiting the biological specificity of the cohort.

In our population, most patients with autoimmune comorbidities were affected by Hashimoto’s thyroiditis (HT), one of the most common organ-specific autoimmune diseases in Sardinia (65). However, studies investigating the association between sHLA-G levels and HT remain limited and often report conflicting results (66, 67), making it unlikely that HT alone significantly influenced the findings.

Importantly, as shown in **Supplementary Figure S2**, the comparison of sHLA-G levels between patients with and without additional autoimmune conditions did not reveal any statistically significant differences. In addition, previous research has indicated that the presence of extrahepatic autoimmune diseases does not appear to negatively impact the long-term clinical outcomes of PBC (64). One additional aspect to consider is the absence of immunohistochemical data on liver tissue, which prevents the direct evaluation of HLA-G expression in infiltrating immune cells. Future studies integrating tissue-level analyses will be crucial to validate and further explore the mechanisms proposed in this work.

Prior research has thoroughly examined the role of HLA-G in Sardinian patients with AIH-1, showing a significant correlation between lower sHLA-G levels and increased disease severity (38). This suggests that, in addition to its role in AIH-1, HLA-G may also play a significant role in PBC. However, the mechanisms by which the HLA-G*01:01:01:08/UTR-1 haplotype and low sHLA-G levels influence disease onset, therapy response, and progression require further exploration. In conclusion, further studies in diverse populations are essential to validate these findings.

The levels of sHLA-G, particularly those observed in individuals carrying the HLA-G*01:01:01:08/UTR-1 haplotype, could serve as biomarkers for disease severity and therapy response in PBC. This could lead to more personalized treatment strategies, tailoring therapies according to genetic risk profiles.

## Data Availability

The datasets presented in this study can be found in online repositories: https://www.ncbi.nlm.nih.gov/bioproject/PRJNA1191844.
