# Supplementary material for: The role of HLA-G in primary biliary cholangitis and response to therapy
Source: Front Immunol. 2025 Jul 29;16:1585535. doi: 10.3389/fimmu.2025.1585535 (PMC12339529; doi:10.3389/fimmu.2025.1585535)
Supplement: Supplementary file 1 [file DataSheet1.pdf]

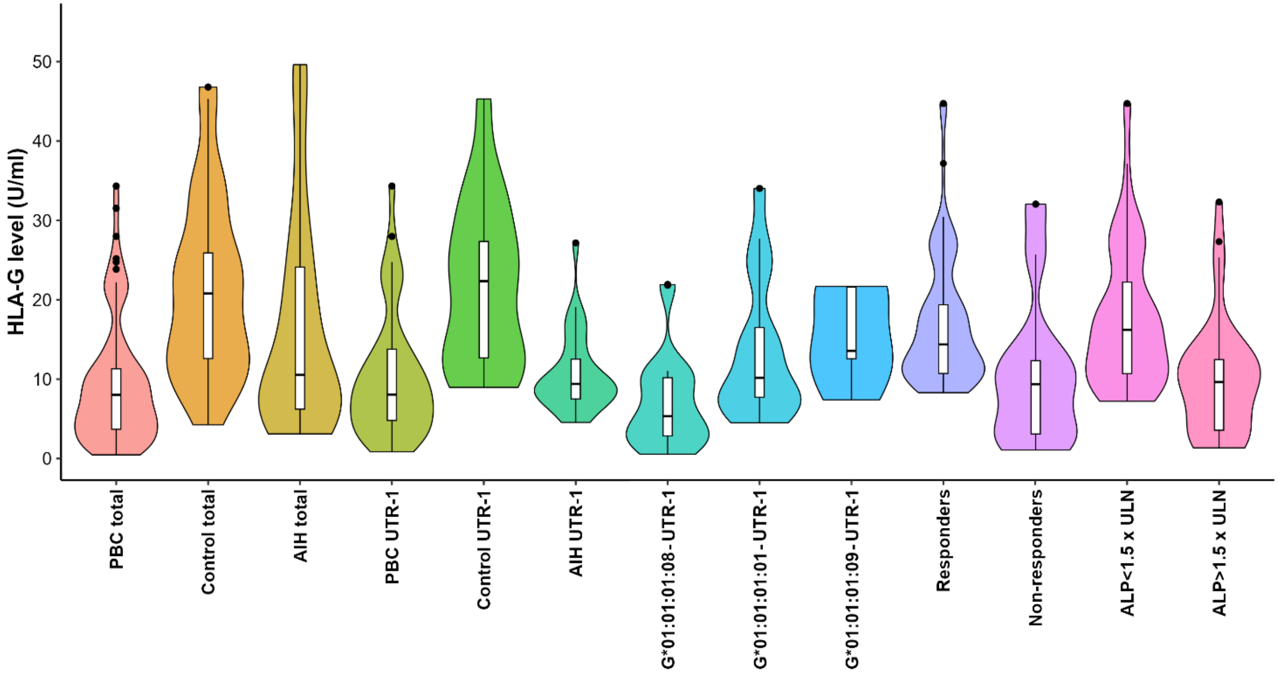

Supplementary Figure S1. *Distribution of sHLA-G plasma levels across different patient and control subgroups. Violin plots illustrate the distribution of soluble HLA-G levels (U/mL) in the following groups: total PBC patients, healthy controls, and AIH-1 patients; PBC, control, and AIH-1 subgroups carrying the UTR-1 haplotype; PBC patients carrying specific HLA-G alleles (G*01:01:01:08-UTR-1, G*01:01:01:01- UTR-1, G*01:01:01:09-UTR-1); therapy responders and non-responders based on UDCA treatment; and PBC patients stratified by ALP levels (<1.5× ULN vs. >1.5× ULN).*

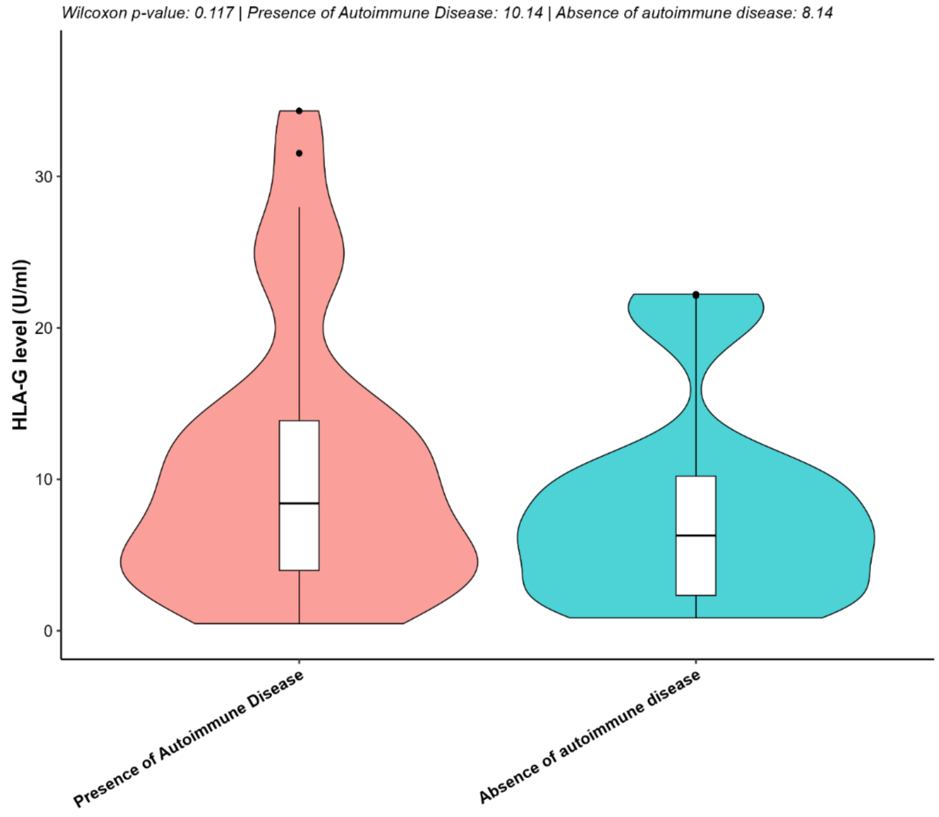

Supplementary Figure S2. *Distribution of sHLA-G plasma levels in PBC patients with or without a coexisting autoimmune disease. Soluble HLA-G levels (U/mL) were measured in patients with a documented diagnosis of additional autoimmune conditions [Hashimoto's thyroiditis (25/58 (0.43%)) polyautoimmunity (16/58 (0.28%)), Coeliac disease (5/58 (0.9%), Sjogren syndrome (4/58 (0.7%)), rheumatoid arthritis 3/58 (0.5%)), type 1 diabetes (2/58 (0.3%)), Ashma (2/58 (0.3%)), Neuromyelitis (1/58 (0.2%))] and compared to those without comorbid autoimmune disorders. P-values were calculated with Wilcoxon.* Patients with (n = 58) and without (n = 108) coexisting autoimmune diseases [mean (95% CI): 10.14 (8.15 – 11.73) U/mL vs 8.14 (7.05 – 9.22) U/mL respectively; median (IQR): 8.08 (3.70 to 13.87) U/mL vs 6.28 (2.34 to 10.21) U/mL respectively; *P* = 0.117].
